# Correction: Overexpression of *VIRMA* confers vulnerability to breast cancers via the m^6^A-dependent regulation of unfolded protein response

**DOI:** 10.1007/s00018-023-04825-5

**Published:** 2023-07-14

**Authors:** Quintin Lee, Renhua Song, Dang Anh Vu Phan, Natalia Pinello, Jessica Tieng, Anni Su, James M. Halstead, Alex C. H. Wong, Michelle van Geldermalsen, Bob S.-L. Lee, Bowen Rong, Kristina M. Cook, Mark Larance, Renjing Liu, Fei Lan, Jessamy C. Tiffen, Justin J.-L. Wong

**Affiliations:** 1 Epigenetics and RNA Biology Program Centenary Institute, The University of Sydney, Camperdown, NSW 2006 Australia; 2 Faculty of Medicine and Health, The University of Sydney, Camperdown, NSW 2006 Australia; 3 Gene and Stem Cell Therapy Program Centenary Institute, The University of Sydney, Camperdown, NSW 2006 Australia; 4 Victor Chang Cardiac Research Institute, Sydney, NSW 2010 Australia; 5 Shanghai Key Laboratory of Medical Epigenetics, International Laboratory of Medical Epigenetics and Metabolism, Ministry of Science and Technology, Institutes of Biomedical Sciences, Fudan University, Shanghai, China; 6 Key Laboratory of Carcinogenesis and Cancer Invasion, Ministry of Education, Liver Cancer Institute, Zhongshan Hospital, Fudan University, Shanghai, 200032 China; 7 Charles Perkins Centre, University of Sydney, Camperdown, NSW 2006 Australia; 8 School of Clinical Medicine, Faculty of Medicine and Health, UNSW Sydney, Kensington, NSW 2052 Australia; 9 Melanoma Epigenetics Laboratory Centenary Institute, The University of Sydney, Camperdown, NSW 2006 Australia; 10 Locked Bag 6, Newtown, NSW 2042 Australia

**Correction: Cellular and Molecular Life Sciences (2023) 80:157** 10.1007/s00018-023-04799-4

In this article the affiliation details for Author Justin J.-L. Wong were incorrectly process and the funding note in the article contains error. The update affiliation and Acknowledgements is as follows.

The correct author affiliation should be

Justin J.-L. Wong^1,2,7,10^

The updated Acknowledgements should be

**Funding**

Open Access funding enabled and organised by CAUL and its Member Institutions. This work is funded by a program support funding from the Centenary Institute to JJ-LW. JCT is funded by the Cancer Council of NSW (Grant #RG21-07). NP is a recipient of an NHMRC Postgraduate Scholarship, Sydney Catalyst Postgraduate Scholarship and an Arrow Bone Marrow Transplant Foundation Supplementary PhD Scholarship. JCT is a Cancer Institute of NSW Career Development Fellow. RL is the recipient of a National Heart Foundation Future Leader Fellowship.

The original article has been updated.

